# Transcriptional down-regulation of suppressor of cytokine signaling (SOCS)-3 in chronic obstructive pulmonary disease

**DOI:** 10.1186/1745-6673-8-29

**Published:** 2013-10-18

**Authors:** Jochen Springer, Frank R Scholz, Christian Peiser, Q Thai Dinh, Axel Fischer, David Quarcoo, David A Groneberg

**Affiliations:** 1 Allergy-Centre-Charité, Pneumology and Immunology, Charité – Unversitätsmedizin Berlin, Free University and Humboldt University, Berlin D-13353, Germany; 2 Division of Applied Cachexia Research, Dept. of Medicine, Charité – Unversitätsmedizin Berlin, Free University and Humboldt-University, Berlin D-13353, Germany; 3 Department of Hematology and Oncology, Charité – Unversitätsmedizin Berlin, Free University and Humboldt University, Berlin D-13353, Germany; 4 Department of Respiratory Medicine, Medical School of Hannover, Hannover D-30625, Germany; 5 Institute of Occupational Medicine, Social Medicine and Environmental Medicine, Goethe-University, Frankfurt 60590, Germany

**Keywords:** Chronic obstructive airway disease, Lung, SOCS, Cytokine, Airways

## Abstract

**Background:**

Tobacco is a leading environmental factor in the initiation of respiratory diseases and causes chronic obstructive pulmonary disease (COPD). Suppressor of cytokine signaling (SOCS) family members are involved in the pathogenesis of many inflammatory diseases and SOCS-3 has been shown to play an important role in the regulation, onset and maintenance of airway allergic inflammation indicating that SOCS-3 displays a potential therapeutic target for anti-inflammatory respiratory drugs development. Since chronic obstructive pulmonary disease (COPD) is also characterized by inflammatory changes and airflow limitation, the present study assessed the transcriptional expression of SOCS-3 in COPD.

**Methods:**

Real-time PCR was performed to assess quantitative changes in bronchial biopsies of COPD patients in comparison to unaffected controls.

**Results:**

SOCS-3 was significantly down-regulated in COPD at the transcriptional level while SOCS-4 and SOCS-5 displayed no change.

**Conclusions:**

It can be concluded that the presently observed inhibition of SOCS-3 mRNA expression may be related to the dysbalance of cytokine signaling observed in COPD.

## Introduction

Chronic obstructive pulmonary disease (COPD) is currently estimated to be the 3rd most common cause of death in 2020 [1]. The disease is characterized by an irreversible and progressive development of airflow limitation featuring cough, mucus hypersecretion, inflammatory changes and remodeling of the airway wall [2]. Next to bronchial asthma [3,4], asbestosis [5], or tuberculosis [6,7], COPD also plays a major role in the field of occupational and environmental respiratory diseases [8].

COPD is related to tobacco smoke [9,10] and a common feature in the underlying pathomechanisms may be a dysregulation of cytokine signaling [11]. Cytokine signaling events are accomplished by molecules such as SMADs (derived from the Drosophila homologue MAD and the C. elegans homologue SMA [12,13]) or suppressors of cytokine signaling (SOCS) [14]. SOCS molecules are a family of proteins that function as negative regulators of cytokine signaling pathways [14]. Next to the first members of the SOCS family, CIS-1 and SOCS-1, that were identified as negative feedback regulators of the signal transducer and activator of transcription (STAT)-5 pathway [15] and inhibitors Jak family tyrosine kinases, respectively [16], also the molecule SOCS-3 was identified as a potent suppressor of cytokine signaling mechanisms [17].

The expression of SOCS-3 can be induced transiently by a large number of both inflammatory and anti-inflammatory cytokines such as interleukin (IL)-3, IL-6, IL-10 interferon or interferon gamma (IFN-gamma) [18]. It has also been shown that SOCS molecules can potently inhibit the Jak/STAT pathway in various inflammatory diseases including autoimmune arthritis [19] or experimental intestinal inflammation [20].

The inhibition of cytokine signaling via the action of SOCS may also play an important role in the pathophysiology of chronic obstructive airway diseases [21] and a study has shown that SOCS-3 regulates the onset and maintenance of T_H_2-mediated responses in bronchial asthma.

Since there are no data available on the expression of this important cytokine signal inhibitor in COPD, the aim of the present study was to address the transcriptional expression level of SOCS-3 along with SOCS-4 and SOCS-5 in bronchial tissues of a previously characterized cohort of COPD patients [12,22].

## Methods

### Human biopsies

Transcriptional expression of SOCS-3, SOCS-4 and SOCS-5 was assessed in bronchial biopsies of a previously characterized cohort of nine COPD patients [12]. The patients´ mean age was 61 ranging from 52 to 77. All patients did not have atopic diseases but were smokers. COPD was characterized as level II according to the GOLD classification [23,24]. As control group, tissues were obtained from a previously described groups of subjects (n = 7, male and female) who were undergoing routine examinations for bronchial carcinoma without pathology [12]. The mean age was 67 ranging from 50 to 77. Their forced expiratory volume in 1 second (FEV_1_) was over 90% (mean: 102.1%, range: 94.6% to 113%). Bronchial mucosal biopsies were obtained by routine fiberoptic bronchoscopy as described previously [25]. All subjects were free of interstitial lung diseases, tuberculosis, diffuse malignant lung diseases and had not received radiation- or chemotherapy in the past. The study protocol was approved by the local Ethics Committee (Free University of Berlin).

### Tissue morphology

The morphology of the tissues was assessed as previously described using routine histology [26,27]. The biopsies were cryopreserved and cut to cryostat sections using a routine protocol [28,29]. In brief, after an immersion-fixation in Zamboni-solution for 4 hours and consecutive washing steps in phosphate-buffered solution (PBS), cryoprotection using 18% saccharose (1604, Riedel-de Haen AG, D- Seelze) was carried out overnight. Afterwards the biopsies were frozen in liquid nitrogen-cooled isopentane and stored at −80°C. The tissues were then processed to 8–10 μm sections using a cryostat and stained with a routine hematoxylin protocol [30,31].

### RNA isolation and reverse transcription

Total RNA was isolated from the bronchial biopsies as previously described [12]. In brief, the RNAzol (WAK-Chemie, Bad Soden, Germany) method was performed according to the manufacturer’s instructions and reverse transcription was performed with superscript RT after DNase I digestion (both Invitrogen, Karlsruhe, Germany) according to the manufacturers protocols.

### Real-time quantitative PCR

The quantitative assessment of SOCS transcripts was conducted by the use of the ABI Prism 7700 Sequence Detection system and the Taqman PCR Reagent Kit (Applied Biosystems, Überlingen, Germany) according to the manufacturer’s protocols. For sequence-specific detection, established SOCS primer pairs were used (Table 1). An amplification of the human glyceraldehyde-3-phosphate dehydrogenase (GAPDH) gene was carried out as established internal standard. The primers were synthesized by Roth (Karlsruhe, Germany) and the probes by IBA (Göttingen, Germany). The following cycling conditions were used: 50°C for 2 min, 95°C for 10 min, followed by 40 cycles of 95°C for 15 s and 60°C for 1 min. All results are presented δδ-Ct-values.

**Table 1:** Design of human-specific primer pairs

**Primer**	**Sequence**
SOCS 3 forward	5´-GAGGGTTGGAGAAACCTTCC-3´
SOCS 3reverse	5´-GGCATTTCGGTTAACATTGG-3´
SOCS 3probe	5´-ATGCATCACAGCCCTCACTCACTGT-3´
SOCS 4 forward	5´-CTGCGTGAATCCCTACCACT-3´
SOCS 4 reverse	5´-GGATGGAATGGCTGTAGTCG-3´
SOCS 4 probe	5´-CAGTTCTACCTCCTGTGTTGGTGCCA-3´
SOCS 5 forward	5´-ATCGTGCATCGACAGAGACA-3´
SOCS 5 reverse	5´-TACTGGCAGGCTGACTTGTG-3´
SOCS 5 probe	5´-CAGCACTGCCAACTTTCCCAACATT-3´
GAPDH forward	5´-ACGGGAAACCCATCACCAT-3´
GAPDH reverse	5´-CCAGCATCACCCCATTTGA-3´
GAPDH probe	5´-TTCCAGGAGCGAGATCCCGTCAAG-3´

### Statistics

All data was analyzed using Graph Pad PRISM program. The results are expressed as mean ± SEM and tested for significant differences using the one-way ANOVA and Bonferroni`s Multiple Comparison tests.

## Results

### Bronchial biopsies

#### COPD

**Figure 1:**
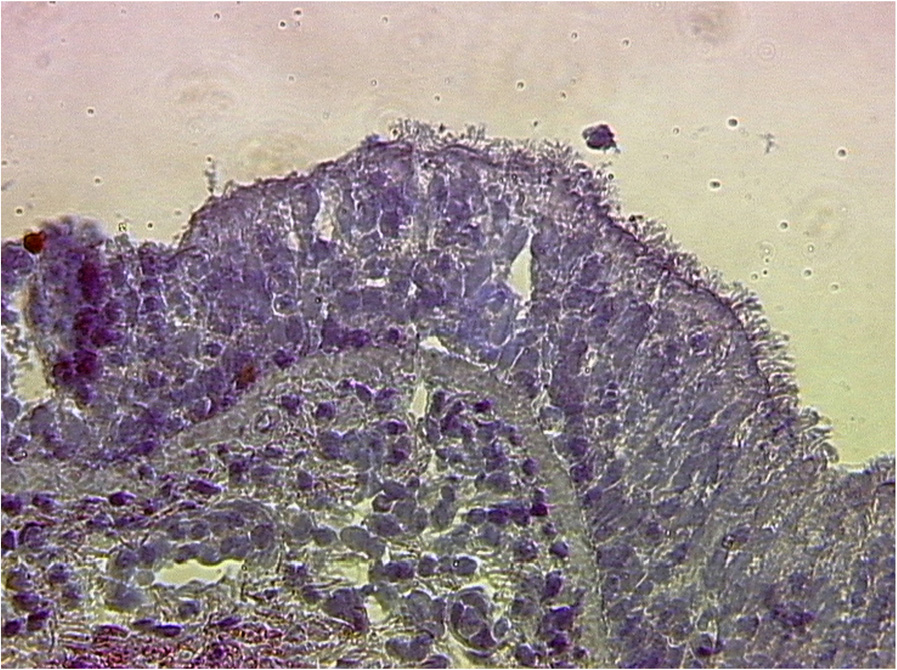
**Morphology of biopsies assessed by hematoxylin staining.** The morphology of the COPD-biopsies was characterized by epithelial hyperplasia and infiltration of inflammatory cells. Original magnification × 400 times.

The biopsies of bronchial mucosal were obtained from nine patients with COPD. They were classified as class II severity according to the GOLD classification and had a FEV_1_ was below 80% of the norm (mean: 70.0%, range: 61.4% to 77.4%), and typical chronic symptoms including cough, dyspnea, and sputum production. The histology revealed chronically inflamed tissues with typical signs of COPD-like lesions. In the submucosal layers, inflammatory cells were present which reached to submucosal glands (Figure 1). Also, single cell necrosis and a loss of ciliae were found.

#### Control

In contrast to the COPD sections, the tissues of seven control subjects were characterized by a regular histological pattern without any signs of inflammatory cell influx or airway remodeling (data not shown).

### Transcriptional SOCS expression levels

The mRNA levels of the different SOCS-3, -4 and 5 that inhibit cytokine signaling were assessed in the COPD tissues and compared to controls. After the presence of all transcripts was shown by qualitative PCR (data not shown), quantitative online PCR was performed in the bronchial biopsies obtained from patients with COPD and healthy controls and significant differences in gene expression were found.

**Figure 2:**
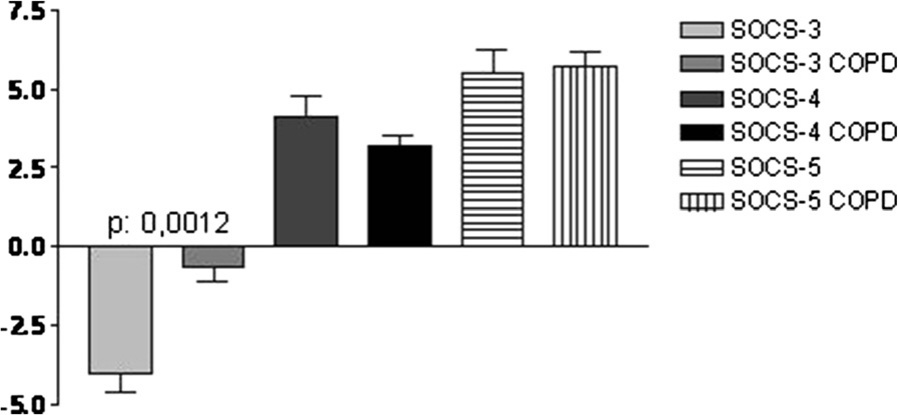
**δδ-Ct-values of the different SOCS in healthy controls and COPD patients, SOCS-3, SOCS-4 and SOCS-5.** **p ≤ 0.01.

While online PCR for the two molecules SOCS-4 and SOCS-5 did not reveal a significant expression difference, the expression level of SOCS-3 significantly differed between the two groups of COPD and control tissues: In controls, the δδ-Ct-values of SOCS-3 mRNA expression was −3,99325 +/− 1,525749 indicating a high level of gene expression. By contrast, in COPD tissues, the δδ-Ct-values were −0,6110268 +/− 1,289377 with a p value of 0.0012 indicating a highly significant downregulation of transcriptional SOCS-3 expression (Figure 2).

## Discussion

A large variety of mediators has been identified which may contribute to COPD pathogenesis [11]. As in allergic bronchial asthma or rhinitis, pro- and anti-inflammatory mediators of inflammation such as tachykinins [32], vasoactive intestinal polypeptide (VIP) [33], histamine [34], nitric oxide [35,36], leukotrienes [37], or opioids [38] and other cytokines [11] are likely to play a role in the regulation of basic pathophysiological mechanisms occurring in COPD. In the present studies, the transcriptional expression of the cytokine signaling inhibiting molecules SOCS-3, SOCS-4 and SOCS-5 was investigated using a established approach of real time quantitative RT-PCR [39] in bronchial mucosal wall biopsies from COPD patients. Transcriptional quantification with the highly sensitive real time RT-PCR was presently chosen and the protocol carried out as previously described. Since the biopsies´ protein contents were not sufficiently high for the performance of western blotting. Previous experiments have demonstrated that the transcriptional expression SOCS-3 is similar to its translational expression [40], indicating that quantitative online PCR represents a valid tool to assess the overall expression level [41].

We found that in COPD tissues, the SOCS-3 δδ-Ct-values were significantly differing from control values indicating a down-regulation in the state of COPD.

Recently, a study has focused on the effects of Fluticasone propionate (FP) and Salmeterol (SAL) on SOCS expression since they are commonly used in combination therapy for patients with COPD [42]. They evaluated the effects of FP/SAL and tobacco smoke (TS) on SOCS-3 in bronchial airway epithelial cells (BAEpCs) which were exposed to TS and subsequently treated with FP or SAL alone or in combinations in the presence and absence of mitogen activated protein kinase (MAPK) inhibitors for either Erk1/Erk2, or p38 or PI3 kinase [42]. In BAEpCs, TS induced IL-6 expression via ERK1/ERK2 MAPK pathway and FP/SAL inhibited TS mediated IL-6 expression. Interestingly, TS downregulated the SOCS-3 expression [42]. This is parallel to our present findings in COPD tissues. The downregulation was mediated via the activation of Erk1/Erk2, and p38 MAPK signaling. When TS exposed BAEpCs were treated with FP/SAL SOCS-3 expression was normalized. Also, FP/SAL combinations induced significantly higher expression of SOCS-3 in BAEpCs when compared to the individual drugs [42].

This transcriptional down-regulation presently observed for COPD might have an impact on the balance of cytokines that determine general immune responses and the onset of T_H_1- and T_H_2-mediated effects. A hallmark study focused on the expression and function of SOCS-3 in allergic bronchial asthma since the functional relevance of SOCS-3 in the allergic, T_H_2-mediated immune response was not clear [43]. It was shown that the expression level of SOCS-3 was increased in asthma and correlated with the pathology of this T_H_2-mediated allergic disease. Since the T cell-constitutive expression of SOCS-3 in an animal model led to an increase in airway hyperreactivity it was suggested that a T_H_2-specific expression of SOCS-3 plays an important role in the disease [43] and that SOCS-3 may not only be a marker for allergic diseases but may also represent a novel therapeutic target.

In contrast to the increased expression in bronchial asthma, we here found a transcriptional down-regulation of SOCS-3 in COPD. In this respect, there are major differences in the cellular inflammation between COPD and asthma. While mast cells and eosinophils play a prominent role in allergic asthma, the major inflammatory cell types in COPD are macrophages and neutrophils [44-46] and an increased sputum neutrophilia is related to an accelerated decrease in FEV_1_ and more prevalent in COPD patients with chronic cough and sputum production [47]. Lymphocytes are also involved in inflammatory mechanisms underlying COPD [48,49] but the lymphocyte repertoire differs to a large extend if compared with asthma. Increased numbers of CD8-positive T-lymphocytes are found in the airways of COPD patients [44-46] and the degree of airflow obstruction correlates with their numbers [50] in contrast to allergic asthma, which is characterized by increased numbers of CD4-positive T-lymphocytes [51,52].

Similar to these differences in inflammatory cell populations that was demonstrated for asthma and COPD in the past years, a different expression pattern of cytokines and cytokine signaling inhibitors may be present in asthma and COPD. To this extend, we here shown that SOCS-3 is transcriptionally downregulated in COPD and therefore shows an expression pattern in COPD reciprocal to that in asthma, in which the molecule was shown be upregulated [43].

A further allergic disease was also characterized to have an expression level of SOCS-3 contrary to the presently identified COPD profile [41]. It was shown elevated mRNA levels of SOCS-3 and GATA-3 are present in PBMC of patients with atopic dermatitis. In contrast to GATA-3 mRNA levels which were normalized after a successful therapy, the levels SOCS-3 did not change [41].

It would be interesting to study the functional role of SOCS-3 using an animal model of experimental COPD and different approaches to mimic COPD have been developed in the past but are limited in comparison to models of allergic asthma since they usually do not mimic all major features of human COPD.

Depending on the duration and intensity of exposure, noxious stimuli such as tobacco smoke, nitrogen dioxide, or sulfur dioxide could be used to induce signs of chronic inflammation and airway remodeling wile emphysema could be achieved by combining such an exposure with the instillation of tissue-degrading enzymes. However, this such studies can not be realized at the moment since mice either constitutively expressing or lacking the SOCS-3 gene have a defect in fetal liver erythropoiesis or placental function, both leading to embryonic lethality [53,54]. In future, conditionally gene-targeted systems may be of help to answer the question of the functional role of SOCS-3 in COPD and modern techniques such as laser-assisted single/oligo cell analysis [55] may further dissect the impaired SOCS signaling pathway on the cellular level. This should be combined with molecular biology [55,56], histo-/cytochemistry [57-59] and pharmacological [12,35,60] techniques.

In conclusion, the present studies revealed a direct link between COPD and alterations in the transcriptional regulation of SOCS-3 that was demonstrated to play a major role in bronchial asthma. The present results indicate that the regulation of SOCS may differ in COPD compared to asthma and suggest that these cytokine signaling inhibitors also play a role in pathomechanisms underlying the inflammatory changes in COPD.

## Competing interests

The authors declare that they have no competing interests.

## Authors’ contributions

JS, FS, CP, QTD, DQ, DAG have made substantial contributions to the conception and design of the study, acquisition of the data and interpretation. They have been involved in drafting and revising the manuscript. All authors have read and approved the final manuscript.
